# Stress sources and coping strategies in medicine students

**DOI:** 10.1192/j.eurpsy.2023.1892

**Published:** 2023-07-19

**Authors:** A. Bosun, R. Kalinovic, C. Munteanu, A. C. Pascariu, G. Vlad, V. R. Enatescu

**Affiliations:** 1 Psychiatry, “Pius Brinzeu” Emergency County Hospital-Psychiatric Clinic; 2 Genetics; 3 Biochemistry, “Victor Babes” University of Medicine and Pharmacy, Timisoara, Romania; 4 Department of Automation and Applied Informatics, “Politehnica” University of Timisoara, Faculty of Automation and Computers; 5 Psychiatry, “Victor Babes” University of Medicine and Pharmacy, Timisoara, Romania

## Abstract

**Introduction:**

Medical school can be highly stressful and demanding, and negatively impact the well being of the students. Identifying sources of stress and a better understanding of the ways medical students cope can be helpful towards finding ways to increase the quality of life and efficiency of future physicians.

**Objectives:**

We sought to assess what the main sources of stress and coping strategies are for medical students, as well as how year and socio demographic factors like gender influenced the coping strategies used.

**Methods:**

We have performed a cross-sectional study on 489 medical students from Romania that have been asked to complete a survey which included the most common sources of stress, as well as the COPE inventory to assess what are the strategies that students use for coping with stress. Descriptive and comparative analysis of the data was performed using R software.

**Results:**

Most students have reported stress related to learning and the academic setting. The high volume of material to be learned is by far the greatest source of stress for medical students, followed by weekly schedule and methods of examination. First year students are more preoccupied with accommodation and lack of recreational activities, while, comparatively, sixth year students tend to perceive the academic process itself as more stressful. The coping methods used most by medical students are active coping, planning, and positive reinterpretation of stressful events. Fortunately, the least used way of coping with stress is alcohol / drug use, as well as denial and behavioral disengagement, the latter two being associated with poorer academic performance. Compared to their older colleagues, first year students tend to turn more to religion and denial to cope with stress, while sixth year students show more acceptance and active coping mechanisms. Women are more focused on emotions, and tend to use venting more, as well as using emotional support more often than men, while men turn more to humor and psychoactive substances than women do.

**Conclusions:**

The profile of stress sources and coping strategies of medical students differs by year of medical school and is influenced by socio demographic factors. Certain maladaptive coping strategies may affect an individuals’ academic success, yet most students are using active, problem-focused strategies to deal with stress.

**Disclosure of Interest:**

None Declared

